# Use of plumage and gular pouch color to evaluate condition of oil spill rehabilitated California brown pelicans (*Pelecanus occidentalis californicus)* post-release

**DOI:** 10.1371/journal.pone.0211932

**Published:** 2019-02-27

**Authors:** Deborah L. Jaques, Kyra L. Mills, Barton G. Selby, Richard R. Veit, Michael H. Ziccardi

**Affiliations:** 1 Pacific Eco Logic, Astoria, Oregon, United States of America; 2 Oiled Wildlife Care Network, Karen C. Drayer Wildlife Health Center, School of Veterinary Medicine, University of California, Davis, Davis, California, United States of America; 3 EH1, San Carlos, California, United States of America; 4 Biology Department, College of Staten Island/City University of New York, Staten Island, New York, United States of America; Hawaii Pacific University, UNITED STATES

## Abstract

Sublethal effects of oil spills may dampen seabird rehabilitation success due to lingering negative impacts of contamination and stress on reproduction and long-term survival. These effects can be difficult to measure while birds are in care as well as once birds are released. Expression of sexually selected traits that are sensitive to condition can provide information on physiological status of birds. We evaluated plumage molt and gular pouch skin color of California brown pelicans (*Pelecanus occidentalis californicus)* following oil contamination and rehabilitation to test for differences between previously oiled and rehabilitated (post-spill) and presumably uncontaminated pelicans. Post-spill pelicans released with either color leg bands alone, or bands plus harness-mounted satellite GPS tags, were relocated and visually assessed in the field at non-breeding communal roosts and compared to surrounding unmarked pelicans in the general population. Non-oiled pelicans bearing GPS tags were also included in the study. Post-spill pelicans lagged the general population in molt of ornamental yellow crown feathers but hind neck transition into white plumage was not significantly different. Both post-spill and non-oiled pelicans wearing GPS tags had lower gular redness scores than the unmarked, non-oiled population. Pre-breeding gular pouch redness of post-spill pelicans was more strongly influenced by wearing of a GPS tag than a history of oil contamination and rehabilitation. Gular pouch redness of post-spill pelicans in the first 18 months after release was positively correlated with long term survivorship. If gular pouch color is a condition-dependent sexual signal and overall health influences plumage molt progression, our results indicate that many post-spill pelicans marked with bands alone were in relatively good condition going into the next breeding season, but those released with electronic tags experienced additional stress due to wearing the equipment, introducing a confounding variable to the post-release study.

## Introduction

Wildlife exposed to petroleum products after spill incidents can be negatively affected in multiple ways. The primary acute impact to these animals is from external contamination by oil and subsequent hypothermia, increased metabolic effort, and emaciation [1–4]. Ingestion or inhalation of polyaromatic hydrocarbons can cause liver and kidney damage, compromised immune function, and hormone disruption [2, 5, 6]. Oil-contaminated seabirds may succumb immediately to oiling or may endure several days of exposure leading to death if not rescued and rehabilitated. Capture, washing, handling, and time in captivity can both reverse the effects of exposure or, possibly compound the initial impacts of oiling [7, 8]. Sublethal effects of oil spills on seabirds may result in failure to thrive despite plumage restoration and other rehabilitation efforts [4, 9, 10]. Such impacts are difficult to measure once birds are released but may play an important role in population restoration [11–13].

Researchers have used a variety of techniques to track the fate of oiled seabirds following rehabilitation and release. These include quantifying band recoveries of live and dead birds, monitoring movement patterns with electronic tracking, and documenting reproductive success [9, 10, 14–17]. Electronic tracking methods have improved knowledge of near-term post-spill bird survival [18–20]; however, these devices introduce another variable to post-release studies.

Brown pelicans (*Pelecanus occidentalis*) are a seabird commonly oiled following nearshore spills in the United States. Survival and dispersal of previously oiled and rehabilitated California brown pelicans (*P*. *o*. *californicus*) were examined in the early 1990’s by Anderson et al. [9]. Conventional VHF radiotelemetry and auxiliary color banding were used to track movements and association with breeding colonies up to two years post-release. None of the previously oiled pelicans were known to breed and only 9% were detected alive two years after the spill. Anderson et al. concluded that oiling, and possibly handling stress, resulted in long-term injury to pelicans, including failure to breed, and lower survival than controls. Those results supported the supposition that long term sublethal effects of oiling may preclude restoration of some seabirds to the breeding population. More recently, Miller et al. [21] detected a small number (approx. 2% of those rehabilitated and released) of color banded Eastern brown pelicans (*P*.*o*. *carolinensis)* successfully breeding one year after oiling from the Deepwater Horizon spill. An increasing number of studies have demonstrated that seabird oil spill rehabilitation efforts can succeed in restoring birds to normal function, but further evidence is needed–specifically projects that extend beyond acute survival [22, 23].

Identification of visible external features that indicate physiological responses to contaminant exposure have helped advance the field of ecotoxicology [24–26]. Variation in expression of carotenoid-based colors has been used as a tool for evaluation of contaminant effects and overall condition of birds [12, 27–29], but evaluation of plumage and soft-part colors have not previously been used in studies of post-spill rehabilitated seabirds. Little information is available on impacts of oil exposure, washing, and rehabilitation on seabird molt and plumage condition after release, but endocrine disruption or other stressors that affect body condition could feasibly affect subsequent molt and feather pigmentation. Molt is energetically expensive (up to 5–30% of daily energy budget) and is generally regulated by photoperiod and hormonal cycles [30]. Timing and duration of molt can be influenced by factors such as age, sex, reproductive and nutritional status, geographic origin, and variable environmental conditions [30–34] complicating application to pollutant studies.

Brown pelicans gradually develop definitive adult plumage over a period of 3–5 years and are sexually monomorphic [34, 35]. Adults have a complex annual molt cycle that results in radically different seasonal appearances of the head and neck [34–36]. Adult head and neck feathers are generally replaced in the late summer-fall as part of the prebasic molt [37]. Faded dark hindneck feathers are replaced with white feathers, creating a gray appearing hindneck during the transition. Around the same time, yellow feathers begin to grow into the white or speckled crown until the crown is completely yellow and the neck is white in definitive basic plumage. As part of the prealternate molt later in winter, dark brown-black feathers return to the hindneck and crest. The crown’s golden hue may intensify during this time, creating dramatic contrast with the dark hindneck during the courtship period. The yellow crown is thus an ornamental feature that is present in both basic (non-breeding) and alternate (breeding) plumage [37, 38]. The crown transitions to white specked with brown during the nesting season and the brown neck plumage becomes faded and worn by the end of the typical chick-rearing period.

Both sexes of California brown pelicans (hereafter ‘pelicans’) develop a red gular pouch prior to the breeding season, when the dull grayish gular pouch blooms with redness proximally [35]. The pouch is multi-layered, including a highly vascular muscle layer and sub-epithelial melanocyte layer [39]. Pouch redness is thought to indicate hormonal activity and breeding readiness [40]. Red gular sac color in the great frigatebird (*Fregata minor)* during the breeding season has been related to a combination of carotenoids, hemoglobin, and increased blood flow [41]. The specific physiological and biochemical mechanisms surrounding display of gular redness have not been described for pelicans but are likely similar to frigatebirds.

Coloration has been found to indicate health and fitness in many species of birds [42, 43]. Carotenoid pigments are acquired by diet and typically give a red, orange, or yellow hue [29, 44, 45], such as the pelican’s pre-breeding crown and gular pouch colors. Expression of carotenoid colors for sexual signaling is thought to have evolved as an honest advertisement of individual quality since there are physiological costs associated with using these valuable antioxidants for display [43, 46, 47]. The outward expression of sexual characteristics can be a particularly sensitive and readily observable indicator of physiological responses resulting from stressful events [24, 48]. Carotenoid-based coloration has been found to have a negative relationship to environmental toxins [28]. For example, Perez et al. [12, 26] provided experimental and field evidence that during the courtship period, the red spot (a carotenoid-based signal) in yellow-legged gull (*Larus michahellis*) beaks was smaller in birds exposed to polyaromatic hydrocarbons compared to those that were not exposed. Similarly, the hue and brightness of skin colors of kittiwakes during the breeding season showed a negative relationship to endogenous pollution levels [49].

We designed a study to test the null hypothesis that post-spill pelicans observed in the wild would not differ visually from conspecifics with respect to their outward physical appearance. Here, we provide a field-based comparison of molt progression in the head and neck, and expression of redness in the gular pouch between previously oiled/rehabilitated pelicans and the general, presumably never oiled, wild population. We further report on unexpected differences between post-spill pelicans bearing electronic tags versus post-spill birds released with only leg bands, and the relationship of gular color to known survival amongst rehabilitated birds.

## Materials and methods

To further improve estimates of seabird recovery following oiling, the Oiled Wildlife Care Network (OWCN), a program led by the School of Veterinary Medicine at the University of California at Davis, initiated a study to track rehabilitated brown pelican movement patterns and survival following the May 2015 Refugio Beach Oil Spill near Santa Barbara California using Global Positioning Satellite platform transmitter terminal (GPS-PTT) tags [50]. We developed this field investigation as a companion to the remote tracking project. We assumed that adequate numbers of post-spill pelicans could be located with the aid of recent position data from the transmitters and that measures of field observable characteristics, including plumage transitions and gular pouch color, could provide an independent measure of post-spill pelican health in the wild. Non-breeding brown pelicans spend much of their daily time budget onshore at traditional coastal communal roosts [51, 52] where, in certain settings and with appropriate techniques, they can be observed closely without causing disturbance. Surveys at breeding colonies were not feasible for this study due to potential disturbance and remote nesting locations [53].

**Fig 1:**
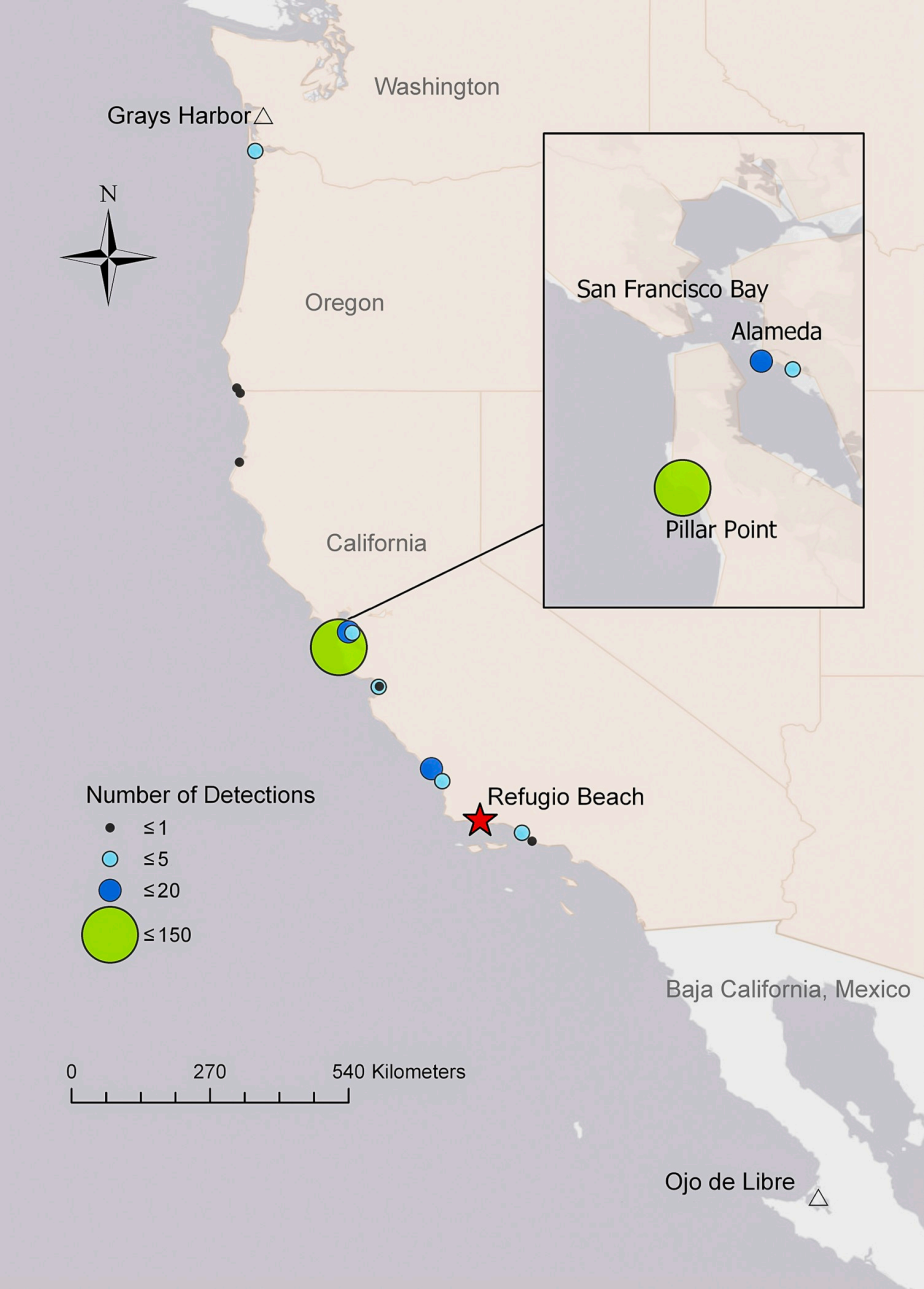
Refugio oil spill site and distribution of post-spill pelican field sightings. Locations of Refugio spill green banded pelican sightings from August 2015 through November 2016 are shown (N = 190 sightings). North and south limits of field surveys are indicated by triangles. Base map credit: Esri, HERE, Garmin, OpenStreetMap contributors, and the GIS users community.

**Fig 2:**
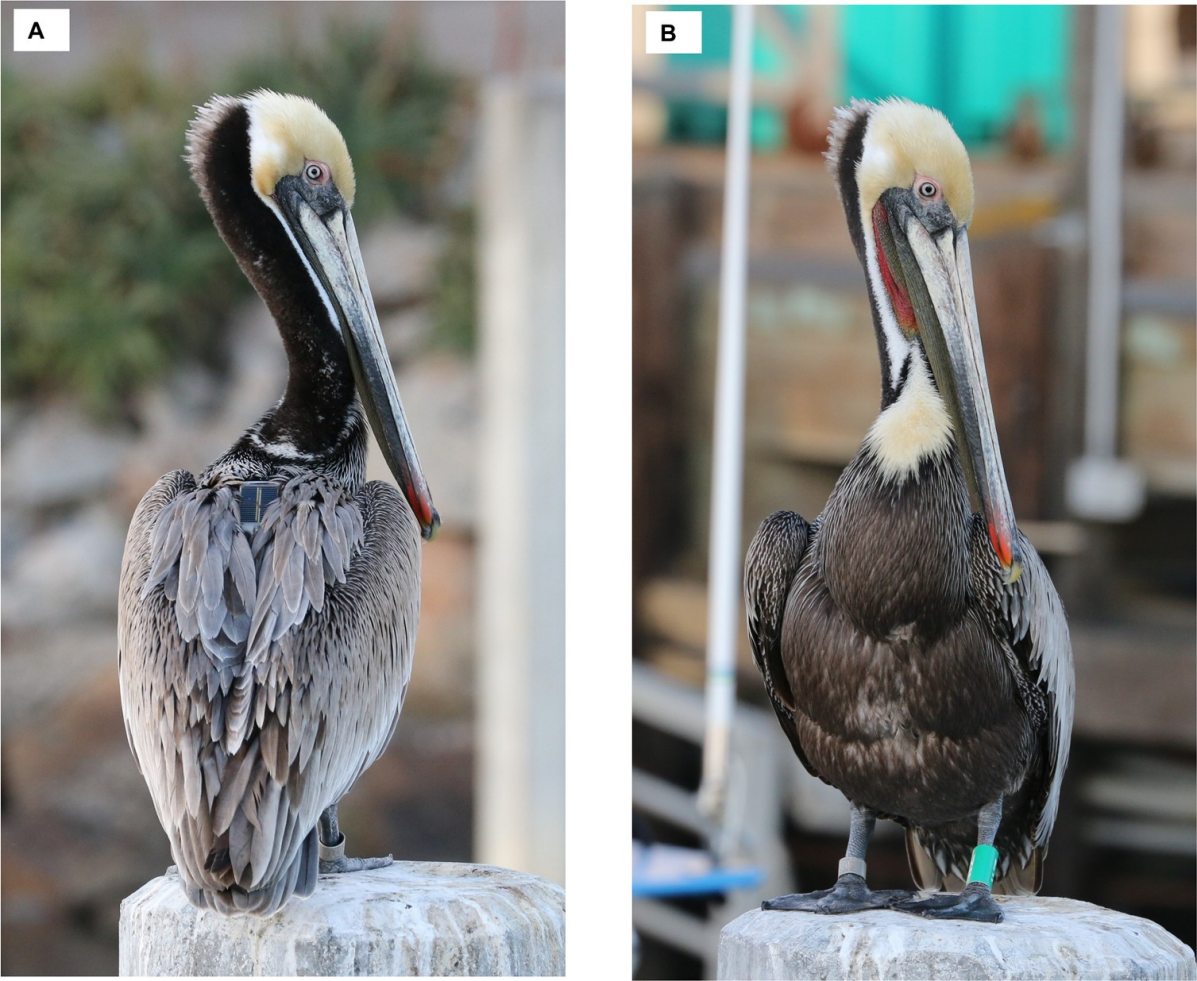
Configuration of GPS-PTT tags on brown pelicans observed in the field. Dorsal (A) and ventral (B) views shown on Z11, 17 March 2016, after about 9 months of wearing the equipment.

The Refugio Beach Oil Spill occurred on 19 May 2015, in Santa Barbara County, California, USA at 34.46° N, 120.09° W (Fig 1). The spill affected over 100 miles of coastline with oil sheen and tar balls persisting on the water for several weeks. Brown pelicans contaminated with crude oil from the spill were captured with hand nets 1–10 days after the initial event. The extent of oiling on individuals in the study was variable (2–100%) and the duration of exposure was unknown but ranged from 2 to as many as 15 days. The birds were washed, rehabilitated and released approximately 3–5 weeks after oiling just outside the spill zone [54]. All released pelicans (N = 42) were outfitted with aluminum federal bands and field-readable green color leg bands. Thirty-three of the birds were aged as adult based on typical after third year (ATY) plumage [34, 35]. Of these, a subset of 12 adults were fitted with GPS-PTT devices (65g solar units from GeoTrak Inc, Apex, North Carolina). Transmitters were attached backpack style with Teflon ribbon harnesses (Fig 2) [50, 55]. Eight additional adult pelicans that did not appear to have been directly exposed to oil were captured in early July 2015 by baiting near the spill site and fitted with the same GPS-PTT tags for comparative studies of movement patterns. These control birds were released within hours of capture. Electronic tagging equipment and procedures are further described in Lamb et al. [56].

Work was completed in accordance with all appropriate State, Federal and university regulations and policies including the U.S. Geological Survey Bird Banding Laboratory with Federal Master Bander permit #23539, the U.S. Fish and Wildlife Service Scientific Collection Permit #MB191637-0, and the California Department of Fish and Wildlife Scientific Collection Permit #SC-003855. All procedures were conducted under University of California at Davis Institutional Animal Care and Use Committee Protocol #18823. Brown pelicans are protected under the Migratory Bird Treaty Act.

Field reconnaissance was initiated approximately three months after release of the previously oiled birds. Data collection on plumage and color comparisons took place primarily from September 2015—March 2016. Field work occurred in seasonal sessions of 7–10 days and ranged along the coast from Grays Harbor, Washington, USA (46.54°N, 124.06°W) to Ojo de Liebre, Baja California Sur, MX (27.50°N, 113.57°W; Fig 1). Permission to access Naval Base Ventura County lands was granted by Martin Ruane (U.S. Navy) and access to Año Nuevo Island Reserve was provided by Patrick Robinson (U.C. Santa Cruz). All other field locations were open to the public. Pelican roost site atlases were used to help plan search effort within a region and surveys took place from both ground and small boats. The basic survey strategy was non-intrusive searches and observations of post-spill and control transmitter pelicans at non-breeding communal roosts and fish handling areas, with initial effort focused on regions where GPS positions indicated past occurrence of electronically tagged birds. After transmissions ceased and/or these birds moved out of the study range, search effort was focused on large roost sites where leg-banded pelicans were most likely to be detected. For the duration of the study, another observer conducted routine kayak-based searches for banded pelicans at major harbor breakwater roosts in Central California nearly weekly, without consideration of the electronic tracking data. Both observers photographed post-spill pelicans with high resolution DSLRs and image stabilizing telephoto lenses.

Comparisons to the general population were made at the same place and time by evaluating the five nearest neighbors surrounding a marked focal bird. The ‘general population’ consisted of unmarked, presumably never oiled or rehabilitated pelicans. Focal birds included any individual in one of the following three groups; 1) previously oiled pelicans released with color band-only, 2) previously oiled pelicans with band plus GPS-PTT, and 3) non-oiled pelicans with band and GPS-PTT. Plumage aspect and soft-part color characteristics were based on classifications in Schreiber et al. [35].

**Fig 3:**
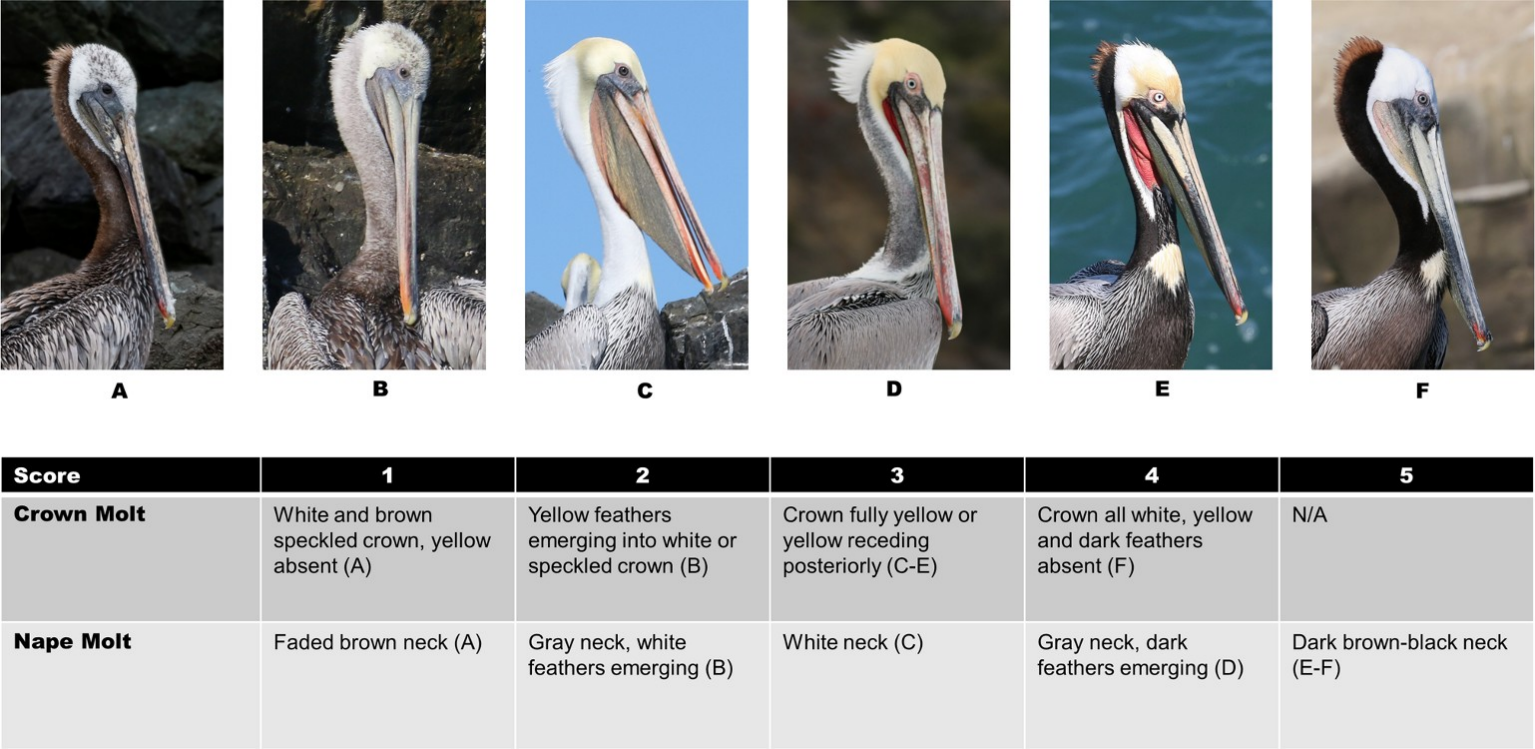
California brown pelican crown and nape index scoring system. The scores shown were the numbers used for the crown and neck molt indices (CMI and NMI).

**Fig 4:**
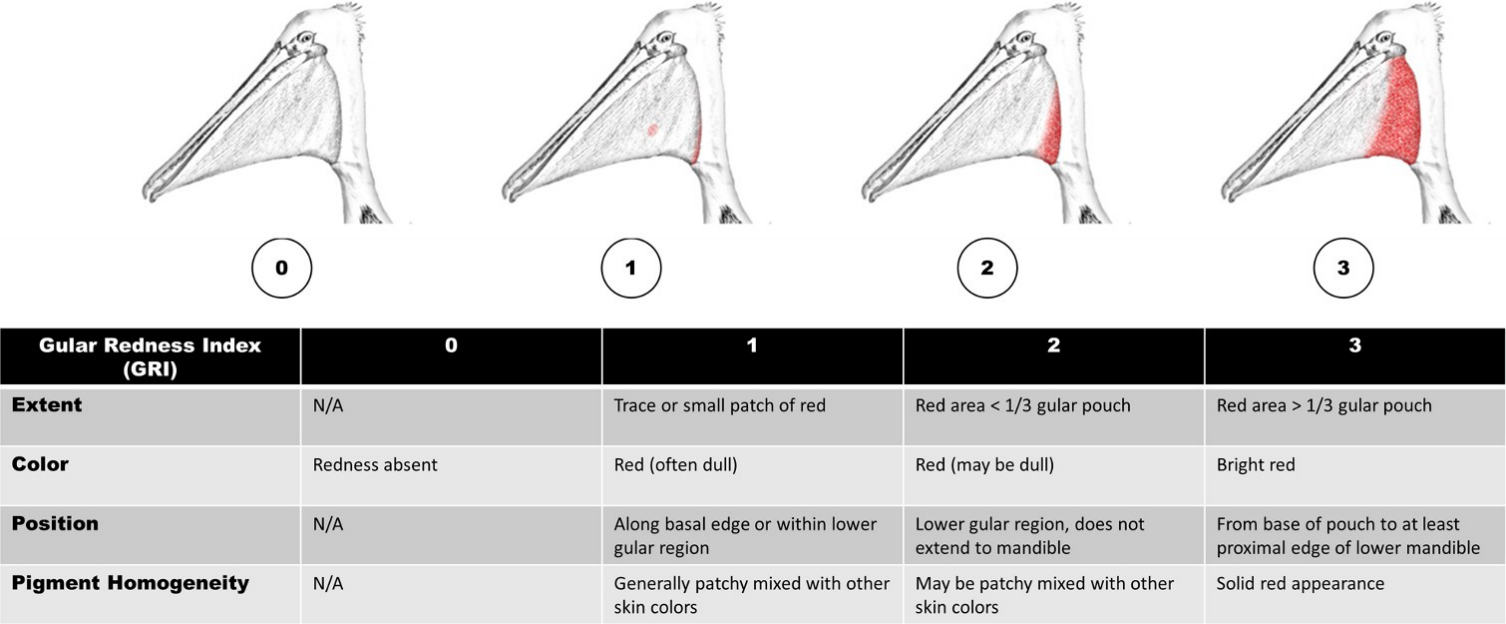
California brown pelican gular pouch scoring system. The gular redness index (GRI) was based primarily on extent of red; other characteristics that typically accompany each score are also included here.

Molt phase and color assessments were limited to adult birds and focused on three distinct and readily visible body parts—the hind neck, the crown feathers, and the gular pouch. These features were visually scored using a combination of direct field observations and digital photographs. The hind neck and crown were categorized as either in a definitive plumage aspect or transitioning between states [37]. An ordinal grading system was developed (Fig 3) resulting in five different potential scores for the neck molt index (NMI) and four for the crown molt index (CMI). We did not attempt to discern differences in yellow crown feather hue but simply the occurrence of yellow feathers. Data for crown and neck analyses were restricted to September 15-December 15, 2015. A third ordinal grading system, the gular redness index (GRI) was based primarily on visual estimation of spatial extent of red in the pouch (0–3) using data collected during September 15-March 21, 2016 (Fig 4). Plumage and color were scored only by the primary observer to reduce potential bias. Occurrence of wetted plumage and shivering was also noted in the field, which can be normal in some circumstances but can also indicate structural failure of plumage and a need to produce endogenous heat [57].

Surveys of banded pelicans to document survival continued through the summer of 2018 and each post-spill bird was photographed. A separate analysis of post-spill survival of the Refugio incident pelicans, incorporating demographic and biomedical factors, is planned [54].

Statistical analyses were conducted using R package v. 3.5.1(R Core Team 2018) [58]. Pelicans were assigned to four different groups as follows: post-spill rehabilitated birds without GPS-PTT tags; post-spill birds with GPS-PTT tags, non-oiled pelicans with GPS-PTT tags; and the general population of wild, non-oiled, unmarked pelicans. Loglinear analyses were used to test for interactions between factors. These analyses included a date factor to incorporate variation in plumage and color over the sampling period. Data were binned into three sampling periods as follows (Sep-Oct, Nov-Dec, and Feb-Mar). The effects of oil and GPS-PTT were modeled separately. In addition, the association between highest GRI observed during 2015–2016 and survival of post-spill birds, was tested using Spearman rank correlation. The survival index was the number of days elapsed between release and latest observation in the field, including detections in 2018.

## Results

**Fig 5:**
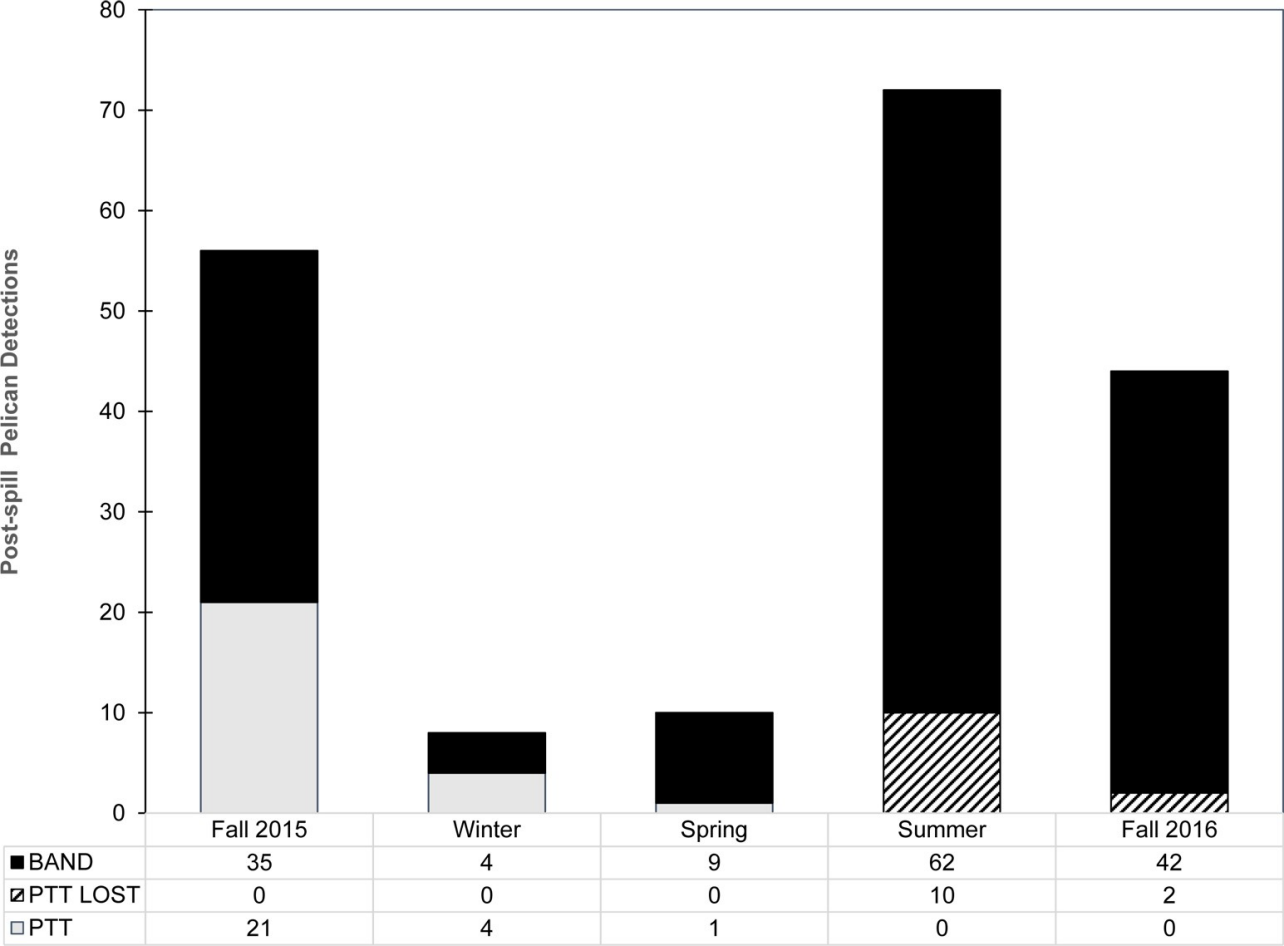
Field encounters of post-spill brown pelicans by season (2015–2016). Data are separated for previously oiled birds released with only leg bands (BAND), equipped with GPS-PTT transmitters (PTT) and birds that shed transmitters but retained bands (PTT LOST). N = 190 sightings of 33 individual post spill birds of all ages.

Most of the Refugio post-spill pelicans (79%) were re-sighted at least once following release. By September 2017, 220 sightings of 33 birds were documented (mean = 6.7 sightings/bird, range = 1–45, SD = 9.2). Frequent surveys of large communal roosts resulted in a greater number of visual encounters of target animals than did attempts to relocate electronically tagged pelicans based on GPS-PTT data feeds showing past positions. Sightings of the post-spill birds were most common in summer and fall (especially July-October) and relatively rare during late winter-spring (Fig 5) when pelicans generally retreat to offshore islands in the breeding range [59]. Tracking data showed that all of the electronically tagged post-spill pelicans moved out of the zone affected by the spill during summer and fall 2015- some as far north as Oregon, and most retreated south into Mexico during winter [50].

Most visual sightings of post-spill birds occurred over 300 km north of the spill region in Central California (Fig 1). This was due to a combination of focused survey effort at two harbor roosts where there were exceptionally large concentrations of pelicans and favorable band-sighting conditions. We counted as many as 2,800 and 3,900 pelicans at two key breakwater roosts, in Pillar Point and Alameda harbors, respectively, during the summer of 2016, while as few as zero occurred in late winter-early spring surveys. Band sightings indicated high turnover of individuals and suggested a well-mixed migratory population, although a few birds were resident to the area during much of the non-breeding season.

Plumage of post-spill band-only pelicans appeared normal with respect to waterproofing and overall integument condition (e.g., none were observed with saturated wet plumage or seen shivering), while four of the 12 pelicans seen wearing transmitters were observed shivering (two post-spill, two control). Plumage disruption around the harness was generally visible around the ventral surface (Fig 2B) and excessive preening around the straps was observed, but not quantified for this study. Ultimately, four pelicans were encountered alive that had shed their transmitters but retained leg bands.

**Fig 6:**
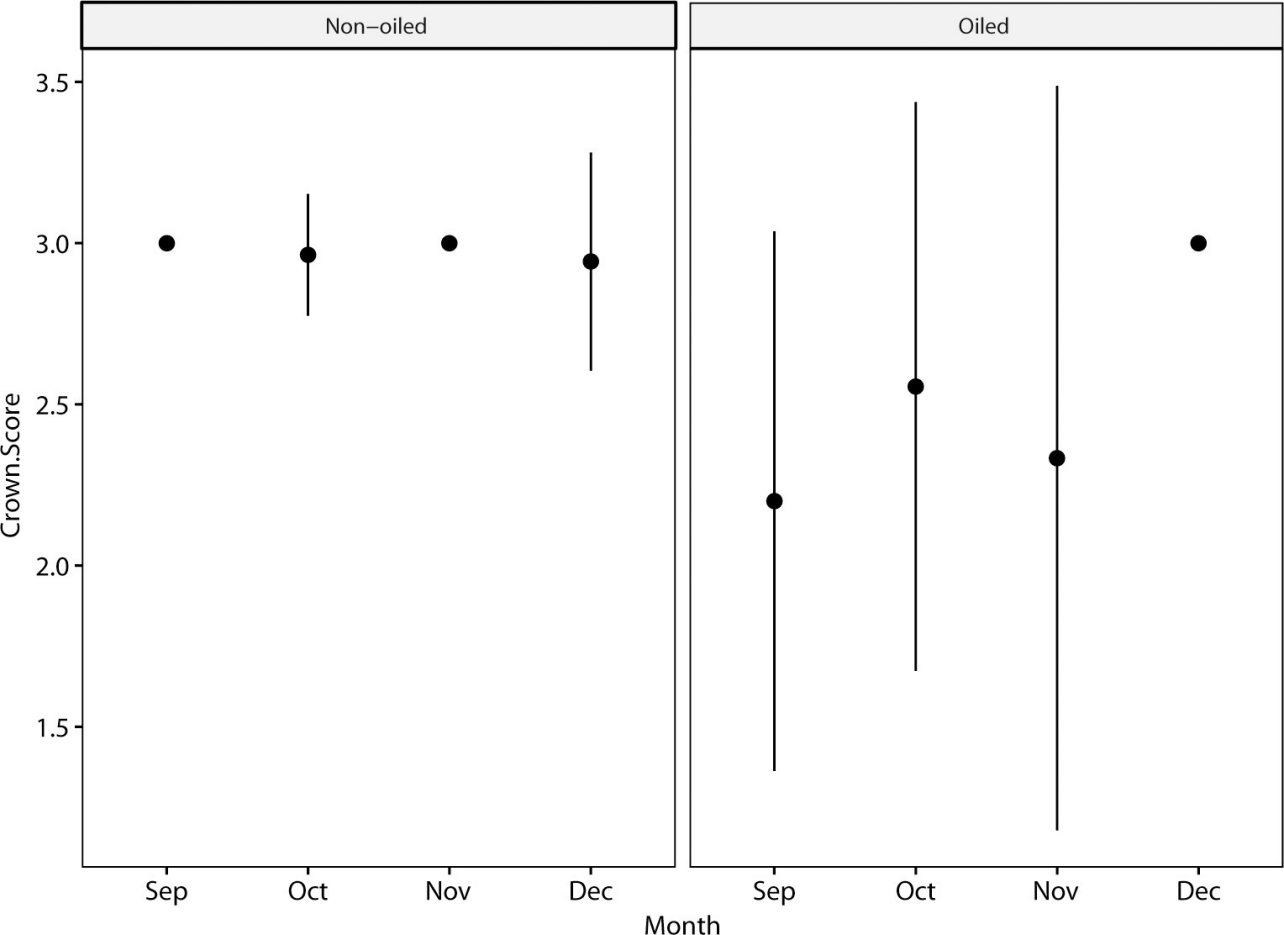
Crown scores compared for non-oiled and oiled pelicans, showing monthly mean and standard deviation. The oiled group includes pelicans released with band-only and GPS-PTT tags (N = 21). The non-oiled group includes only unmarked pelicans (N = 135) and does not include pelicans bearing PTT tags. Data are from September 15-December 15, 2015. Statistical analysis results are presented in Table 1.

During the mid-September—December sampling period, nearly all non-oiled pelicans (98%) displayed solid yellow crown plumage (Fig 3). Emergence of yellow crown feathers was more variable and lagging in the post-spill group (Fig 6); 71% of post-spill birds had solid yellow crowns and the remainder were transitional or entirely lacking yellow feathers. Loglinear analysis incorporating non-oiled pelicans bearing GPS-PTT tags indicated that both previous oiling/rehabilitation and equipment burden significantly influenced emergence of yellow feathers in the crown (Table 1, p < 0.001).

**Table 1:** Results of generalized loglinear analysis of crown molt showing interaction terms.

Variables	Coefficient	Standard Error	Z score	*P*-value
Effect of Oil on Crown				
(Intercept)	-5.3831	2.7926	-1.928	0.0539
Oil	6.0731	1.8366	3.307	0.0009***
CMI	3.1004	0.9368	3.309	0.0009***
Month	-1.3155	1.7672	-0.744	0.4566
Oil*CMI	-2.6741	0.5907	-4.527	<0.0001***
Oil*Month	-0.2747	0.5465	-0.503	0.6153
CMI*Month	0.3513	0.5915	0.594	0.5525
Effect of PTT on Crown				
(Intercept)	-2.5764	2.4887	-1.035	0.3005
PTT	3.7307	1.6149	2.310	0.0209*
CMI	2.2058	0.8381	2.632	0.0085**
Month	-2.6561	1.7756	-1.496	0.1347
PTT*CMI	-2.4798	0.5496	-4.512	<0.0001***
PTT*Month	0.7059	0.5984	1.180	0.2382
CMI*Month	0.7748	0.5953	1.302	0.1931

Oil = previously oiled and rehabilitated (N = 21), PTT = wearing an electronic GPS-PTT tag, both oiled and non-oiled birds (N = 16). The model incorporates non-oiled, unmarked pelicans (N = 135). CMI = crown molt index, Month = data compiled in 2-month bins (Sep-Oct, Nov-Dec). Significance codes:

*** = .001,

** = .01.

* = .05

**Fig 7:**
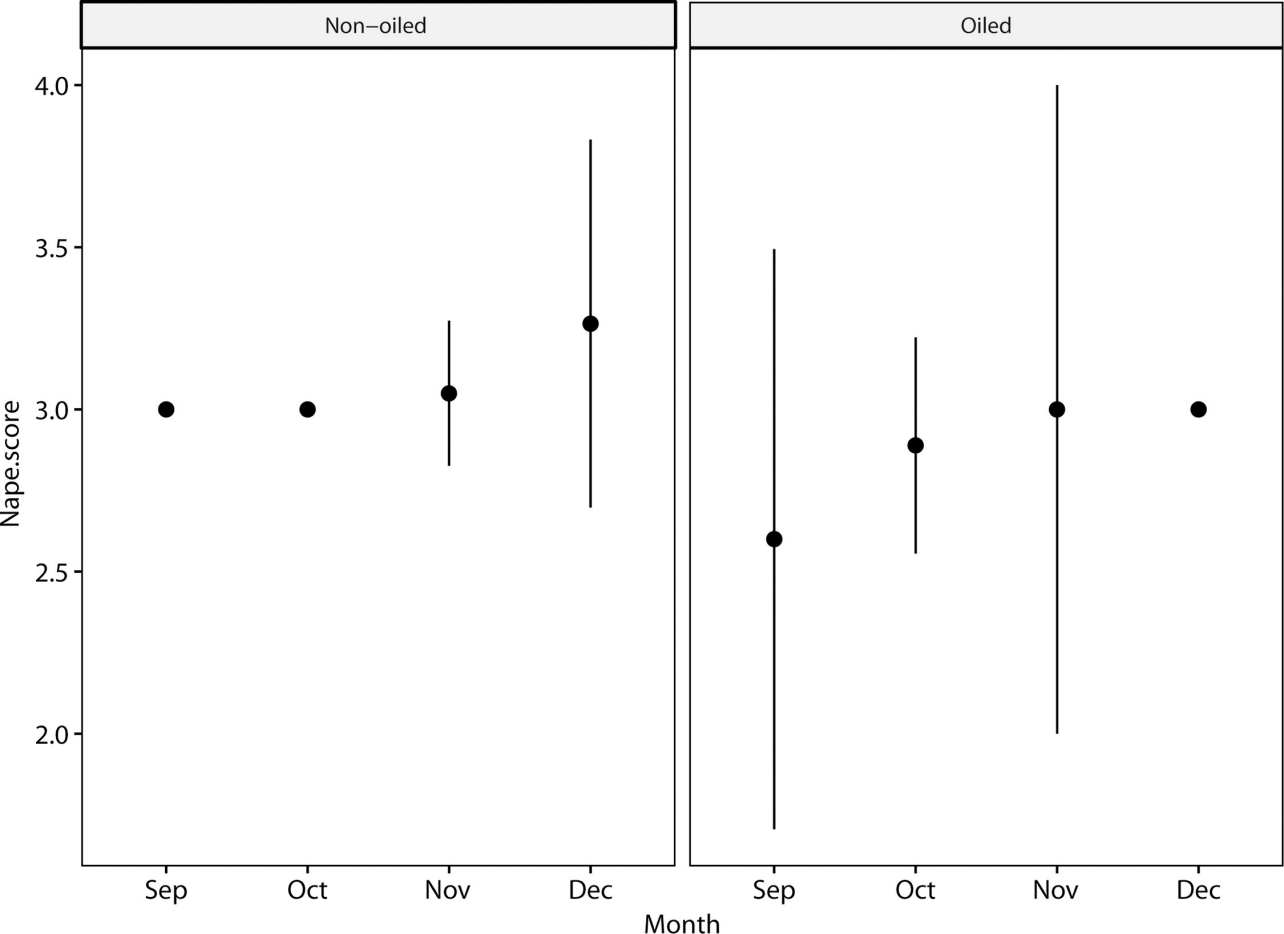
Hindneck scores compared for non-oiled and oiled pelicans, showing monthly mean and standard deviation. The oiled group includes pelicans released with band-only and GPS-PTT tags (N = 21). The non-oiled group includes only unmarked pelicans (N = 135) and does not include pelicans bearing GPS-PTT tags. Data are from September 15-December 15, 2015. Differences in neck molt between groups were not significant according to loglinear analysis (see text).

**Fig 8:**
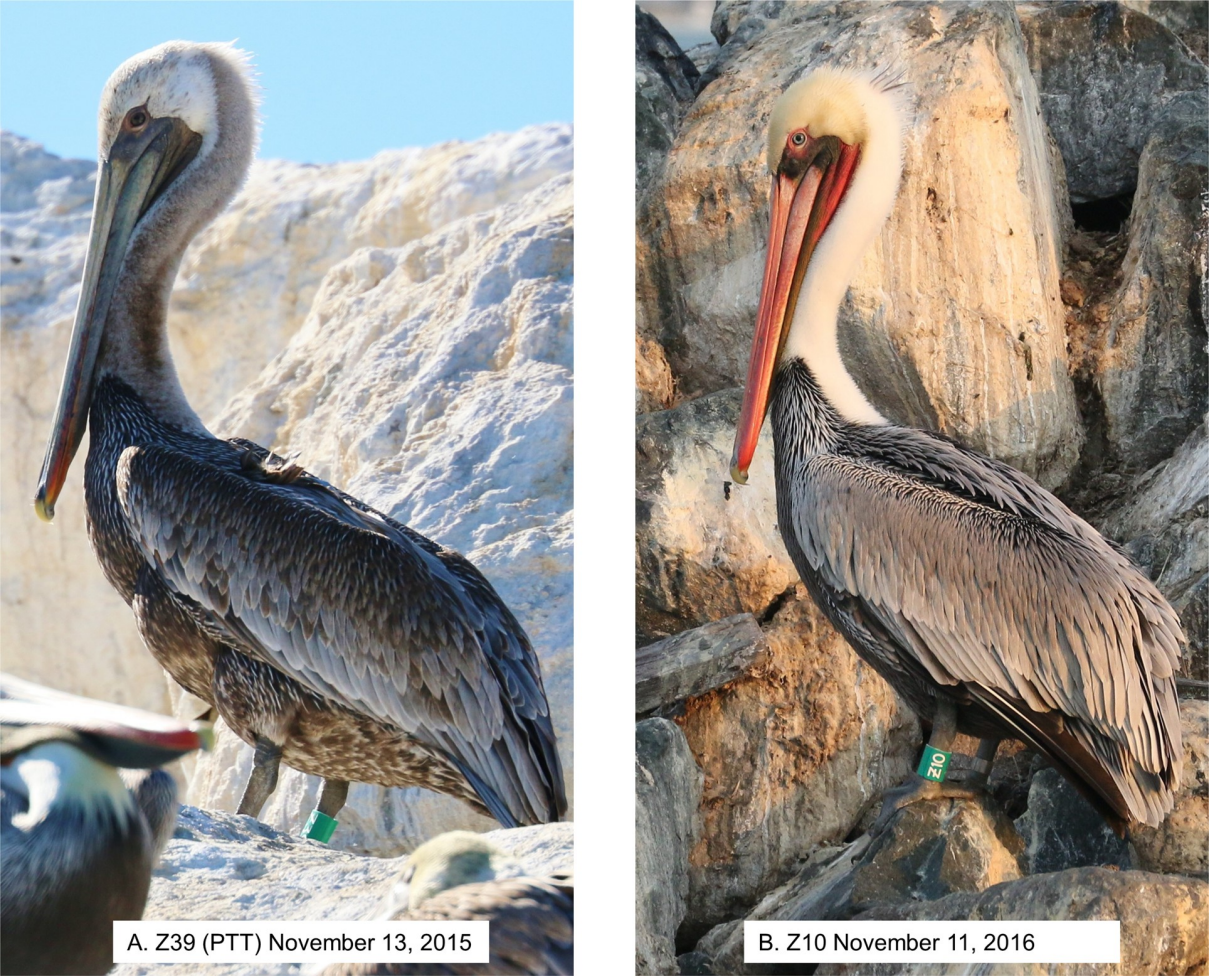
Mid-November brown pelican plumage aspect comparison, lagged versus typical. This example shows lagged molt of a post-spill bird carrying a GPS-PTT tag photographed 6 months after the spill (A) versus seasonally typical molt status in a post-spill bird marked with band-only, photographed 18 months after the spill (B).

Most pelicans (93%) in the non-oiled general population had definitive basic white neck plumage during the sampling period (Fig 3), although some (~ 6%) had progressed further, into prealternate and alternate phase dark hindneck plumage by mid-December. The post-spill group lagged slightly behind the general population in molt of the hindneck, and none of the post-spill birds acquired the dark alternate phase hindneck by mid-December (Fig 7). Replacement of overall body plumage apparently also lagged in some cases but was not quantified (Fig 8). Loglinear analysis indicated that neither previous oiling/rehabilitation or the burden of GPS-PTT tags significantly influenced the molt progression of the neck in this study (Z = -0.65, p = 0.5 and Z = -0.99, p = 0.3, respectively).

**Fig 9:**
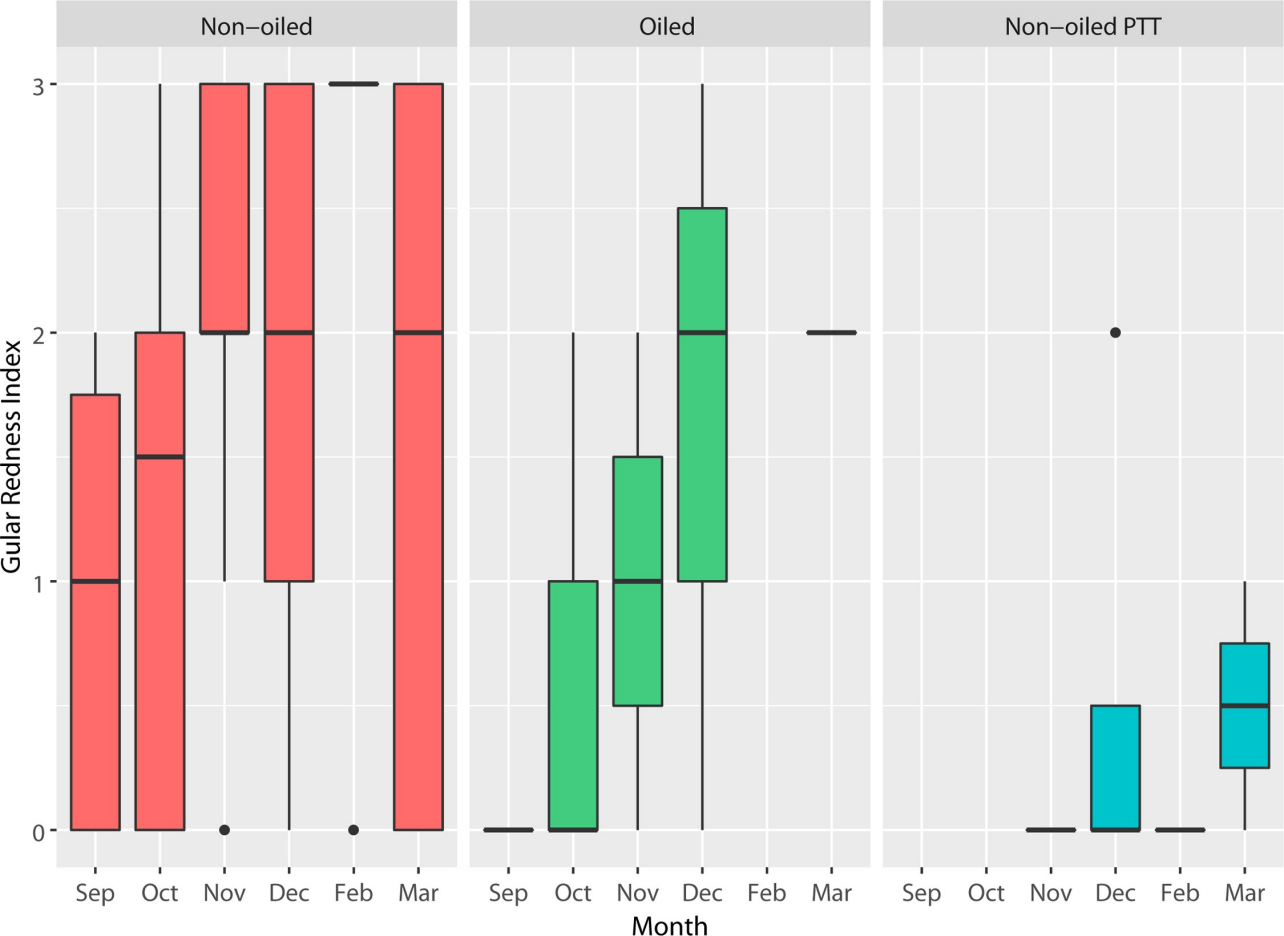
Gular redness scores for non-oiled, previously oiled, and non-oiled GPS-PTT bearing pelicans. Shown are medians, quartiles, and outliers across months. Sample sizes are as follows: non-oiled (N = 135), oiled (N = 17), non-oiled with GPS-PTT (N = 9). Data are from September 15, 2015-March 21, 2016.

Pelicans began to display red pouches at nonbreeding roosts by mid-September 2015, and red pouches were observed through March 2016. Expression of redness in the non-oiled/unmarked population was variable each month within the sampling period but scores tended to be highest in November-February (Fig 9). Previously oiled pelicans and non-oiled GPS-PTT-bearing birds were less likely to express gular redness than the general population. Although both factors (oil and PTT) significantly influenced gular redness according to loglinear analysis (Table 2), the effect of wearing a GPS-PTT was more significant (p < .001). Pelicans wearing electronic tags were more likely to have a GRI score of zero than other groups. For example, in November-December, only 13% of PTT-bearing birds (N = 8) had any discernable trace of pouch redness, while 66% of the post-spill birds released with bands only and 93% in the unmarked non-oiled group showed redness in the pouch (Table 3).

**Table 2:** Results of generalized loglinear analysis of gular pouch redness with interaction terms shown.

Variables	Coefficient	Standard Error	Z score	*P*-value
Effect of oil on gular redness				
(Intercept)	3.1267	0.3276	9.543	< .001***
Oil	-0.7535	0.6441	-1.170	.242
GRI	-0.3883	0.1769	-2.195	.028*
Month	-0.7701	0.1865	-4.129	< .001***
Oil*GRI	-0.5486	0.2633	-2.083	.037*
Oil*Month	-0.47801	0.3994	-1.197	.231
GRI*Month	0.2364	0.0926	2.551	.023*
Effect of PTT on gular redness				
(Intercept)	3.4993	0.3370	10.385	< .001***
PTT	-2.3697	0.6891	-3.439	< .001***
GRI	-0.4878	0.1771	-2.754	.005**
Month	-1.0612	0.2075	-5.115	< .001***
PTT*GRI	-1.5062	0.38446	-3.918	< .001***
PTT*Month	0.85622	0.35109	2.439	.015*
GRI*Month	0.33391	0.09782	3.414	< .001***

Oil = previously oiled and rehabilitated, PTT = wearing an electronic tag (GPS-PTT), GRI = gular redness index, Month = data grouped in 2-month bins (Sept-Oct, Nov-Dec, Feb-Mar). Significance codes:

*** = .001,

** = .01.

* = .05

**Table 3:** Distribution of gular redness scores, separating the effects of previous oiling and wearing electronic tags for loglinear modeling.

			**OIL**			
GRI	*0*	*1*	*0*	*1*	*0*	*1*
3	1	0	22	1	9	0
2	27	2	20	2	3	1
1	12	1	10	0	2	0
0	20	8	9	2	9	0
			**PTT**			
GRI	*0*	*1*	*0*	*1*	*0*	*1*
3	1	0	23	0	9	0
2	29	0	21	1	3	1
1	12	1	10	0	1	1
0	24	4	4	7	7	2
	***Sep-Oct***		***Nov-Dec***		***Feb-Mar***	

Gular redness index scores (GRI) are shown for all previously oiled pelicans (OIL) and those bearing electronic tags (PTT) across binned months, where 0 = no OIL or no PTT, and 1 = previously oiled, or bearing PTT, respectively.

**Fig 10:**
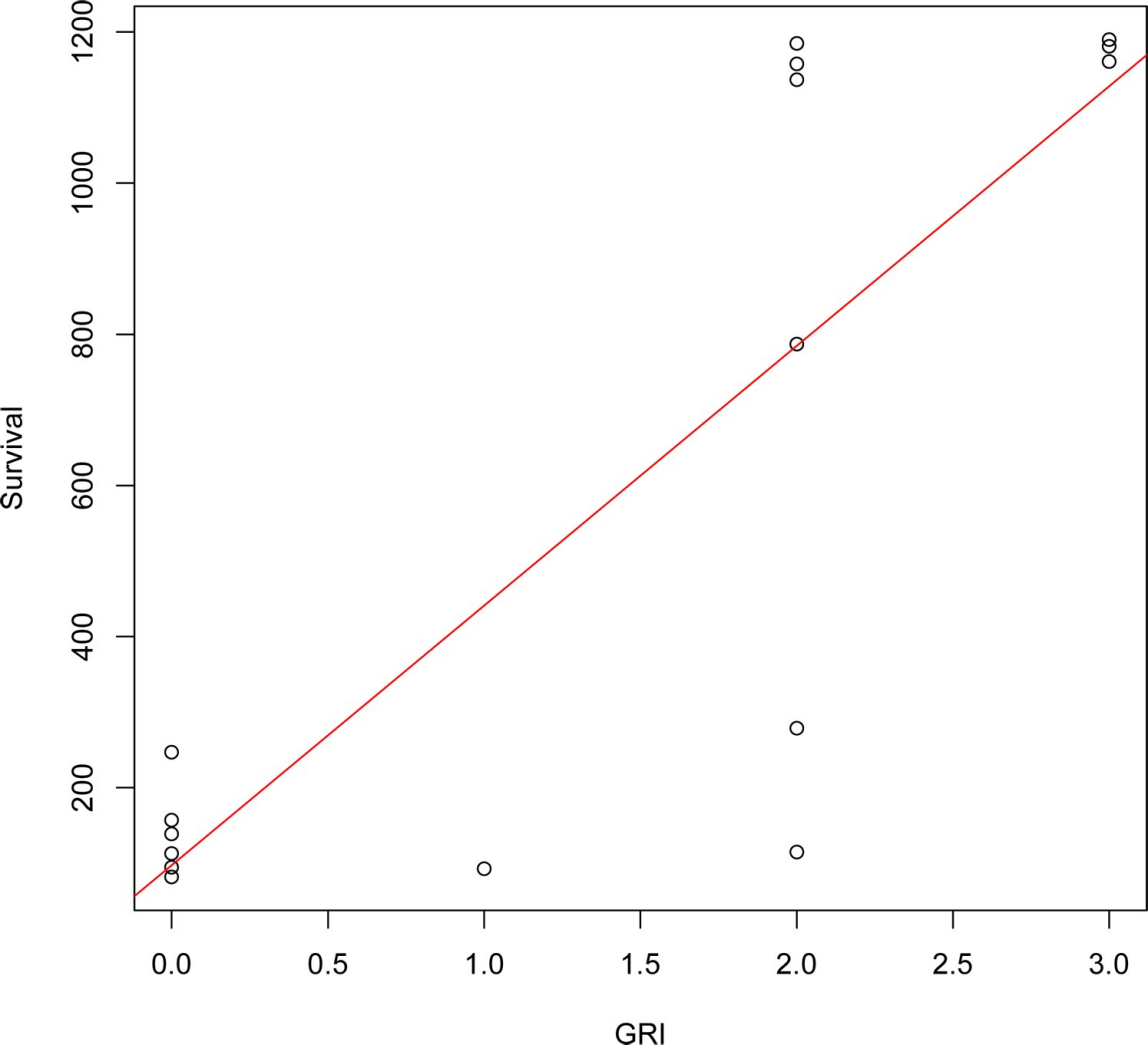
Gular pouch redness index and number of days that previously oiled pelicans were known to survive post-release. Gular redness index (GRI) is the highest score observed in the field (0–3) in the first 18 months after oil contamination; ‘Survive’ is the number of days elapsed between the last sighting of the bird and its release date following rehabilitation. All data (N = 16) are from ATY pelicans. GRI and the survival index were positively correlated (S = 138.7, r_S_ = 0.796, p = 0.0002).

Sightings of banded birds through summer 2018 indicated high survival rates of the Refugio post-spill pelicans overall; at least 60% (25 of 42 pelicans) were alive one year after release and 43% (18 pelicans) were seen alive three years after the spill. Long-term survival of adult post-spill birds was positively correlated with the highest GRI score documented in the first 18 months after oil exposure (Fig 10; S = 138.7, r_S_ = 0.796, p < 0.001). None of the birds with gular scores of zero (no redness in the pouch) were encountered >247 days post-release. Gular redness scores of zero were also associated with known mortality. Of four confirmed pelican mortalities in this study, three were seen alive in the field during the period of expected pouch redness, and all had a score of zero. Three of the pelican carcasses were GPS-PTT-bearing and found by searching in areas where electronic signals suggested lack of normal movement (one previously oiled, two non-oiled). The fourth carcass was from a color band-only bird and found incidentally by a member of the public.

## Discussion

We developed a novel approach to evaluating condition of California brown pelicans in the field following oil spill rehabilitation and release. We found that it was possible to locate adequate samples of post-spill birds without the aid of electronic tracking devices, although it required a lot of survey effort at roost sites that were both heavily used and conducive to close observations. Our results suggest that there were subtle lingering physiological effects of pollution exposure and rehabilitation in the first year after the spill that caused significant differences in crown and gular pouch appearance, contrary to the null hypothesis. Condition-dependent signals represent the sum of environmental pressures on an animal [24]. In this case, the burden of wearing electronic transmitters appeared to be a hindrance to recovery of previously oiled, rehabilitated pelicans and confounded the results of the study. While GPS-PTT tagged birds survived and initially appeared to move normally [50, 56], there was evidently ongoing stress that resulted in a general failure to express gular redness prior to the breeding season for both oiled and non-oiled birds bearing the tags. We recommend that a similar study be repeated without the additional variable of electronic transmitters and with a focus on documenting gular pouch redness in the pre-breeding phase.

Although our results were unexpected with regard to the strong influence of the GPS-PTT tags [56], broad literature reviews have concluded that transmitters negatively affect most aspects of bird behavior and ecology to some degree and may bias resulting data [60, 61]. Kesler et al. [62] found that mallards (*Anas platyrhynchos*) fitted with dummy transmitters attached with Teflon ribbon harness had lower body mass than controls without equipment. Fitted mallards also tended to avoid water, presumably due to greater energetic expenditure to thermoregulate in cold weather and compromised feather insulation caused by the equipment. In our study, one of the equipment-related stressors for pelicans was likely similar bodily heat loss due to plumage disruption around the chest harness and transmitter (see Fig 2B); an issue that may have become aggravated over time.

The fact that there was no discernible difference in plumage quality with respect to waterproofing between previously oiled pelicans marked with leg band-only and the general population was an important finding. Current rehabilitation methods include techniques to ensure that plumage integrity is restored through washing and self-preening and are not released until the birds have met restored waterproofing criteria [63]. This is a major advance compared to the early days of seabird oil-spill rehabilitation, when plumage restoration seemed impossible to achieve [7].

Plumage phase differences between oiled and non-oiled birds could indicate stressors associated with oil exposure, rehabilitation, and/or tracking equipment. A wide range of debilitating sublethal impacts following oil exposure have been previously described [4, 8, 13, 26], but impacts on subsequent feather replacement have not been specified. Our finding that post-spill pelicans moved through the prebasic hindneck plumage transition (from brown to white feathering) at a rate insignificantly different from the general population indicated that this molt process proceeded normally despite the trauma of the spill event.

Sexual signals, including plumage colors that depend on antioxidants such as carotenoids, offer a more sensitive measure of the immunological and nutritional state of animals, since bodily antioxidants tend to be directed to other functions related to survival at the expense of the expression of the sexual traits [64–67]. The less frequent occurrence of yellow crown plumage observed in post-spill pelicans in this study seemed to indicate such a trade-off, but we could not determine if lack of yellow was due to lagging molt or absence of pigmentation in emerged feathers. In addition, brown pelican head rubbing on the uropygial gland may increase the crown’s golden appearance due to adventitious coloring from the sebaceous oils after yellow feathers have emerged [34, 35]. It seems important to note that some of the behaviors we observed in the field suggested that the backpack transmitters may have obstructed normal access to the preen gland by the head, which could result in a less intense yellow hue to the crown and represent another negative impact. Color deficiencies in ornamental plumage may affect mate acquisition and subsequent breeding success [43]. Future field researchers may be able to improve methodology by using calibrated photographs and spectrometers to distinguish variation in the crown’s yellow hue [68].

If one assumes that the red gular pouch provides a positive measure of health in the California brown pelican, our results suggest that, although not equal to the general population, the post-spill birds released with only bands were in relatively good condition going into the first breeding season after the spill, that numerous complex interacting physiological mechanisms were functioning, and that many of these birds could afford the additional costs of color display [46, 69]. Developing and maintaining bright gular coloration in the brown booby (*Sula leucogaster*) apparently incurs oxidative costs for both sexes [47]. Perhaps most importantly, our finding that post-spill pelicans with redder pouches tended to be encountered at a higher rate in subsequent years suggests that gular color may also predict overall survival. Parasite load, nutritional status, and immunocompetence are some of the possible links between survival and color of a dynamic avian integument feature [70–72]. The gular pouch will probably reflect a pelican’s current condition better than plumage color during the pre-breeding season. Dey et al. [73] found that a bare-part ornament was a more reliable status signal than plumage color in a cooperatively breeding bird and recommended an increased focus on bare-parts to expand understanding of animal communication.

Relationships between body condition, integument color, and breeding success are relatively well-known for several seabird species but are not fully understood in the brown pelican. Investigation of the underlying mechanisms surrounding gular pouch color expression and how it relates to pelican fitness would increase the utility of using pouch color in future studies. In the sexually dimorphic great frigatebird, for example, the male displays a bright red inflated throat pouch during courtship that rapidly fades to orange at the onset of incubation. Astaxanthin, a carotenoid pigment, has been found in very high concentrations in the outer dermal layer of the pouch [41]. The spectral pattern of color and the vascularization of the pouch suggests that along with carotenoids, increased blood flow and the presence of hemoglobin may contribute substantially to the overall coloration of the pouch during the display period [41]. Variation in displaying male frigatebird pouch color has been positively correlated to breeding success [74]. The blue and green gular skin color in both sexes of breeding brown boobies is thought to be due to a combination of structural color mechanisms and allocation of carotene pigments [47]. Males expressing greener gular color were found to have increased parental investment in chicks [47, 75]. Kittiwakes in good body condition displayed brighter carotenoid based integument colors around the eye, gape and tongue, than those in poor condition [49]. Pouch color of other brown pelican subspecies varies but none are typically red [35, 36] adding interest to questions surrounding color mechanism and signaling function of the gular pouch in this species.

Targeted field surveys with large sample sizes and from wide geographic ranges are also needed to better establish or update baseline patterns and variation in molt and seasonal color change for California brown pelicans. Schreiber et al. [35] noted that there were many questions surrounding brown pelican molt, including how food supply, region of residence, age, sex, and the interaction between these and other factors, may affect feather replacement; these questions also apply to gular pouch color. For example, we found that gular pouch redness occurred three months earlier than previously described [34, 35]. We saw many birds with red pouches by September at nonbreeding communal roosts, where pouch displays are often used in social interactions [40].

Chronology and outcome of annual breeding effort can affect timing of avian molt [34, 38] but it is not clear how that may have factored into our study results. During the period 2014–2016 there was unprecedented breeding failure throughout much of California brown pelican range due to environmental conditions [76]. Electronic tracking results [50] suggest that pelicans oiled in the Refugio spill included birds from Mexican breeding colonies that had failed or forgone breeding in 2015, and were migrating north along the California coast when caught in the spill. Although pelicans with GPS-PTT tags visited breeding colonies in 2016, none were known to successfully breed [50].

Despite the challenges, field tracking and visual assessment of plumage and gular pouch color of marked brown pelicans has good potential for future ecotoxicology and post-release studies. The expression of gular pouch redness likely plays a role in behavioral ecology at non-breeding communal roosts, as well as breeding colonies, and may provide a valuable window into many aspects of California brown pelican health and fitness. This study also serves as a reminder to use caution when interpreting data based on animals bearing a significant equipment burden and will hopefully help encourage development of improved remote tracking techniques for brown pelicans.

## Supporting information

S1 Fig Crown and nape sampling by bird type, 2015. (PPTX)

S2 Fig Gular pouch sampling by bird type, 2015–2016. (PPTX)

S1 Datafile Data used to test for correlation between gular redness and days of known survival. (CSV)
