# Supplementary figures and images for: Use of plumage and gular pouch color to evaluate condition of oil spill rehabilitated California brown pelicans (Pelecanus occidentalis californicus) post-release
Source: PLoS One. 2019 Feb 27;14(2):e0211932. doi: 10.1371/journal.pone.0211932 (PMC6392258; doi:10.1371/journal.pone.0211932)

## Slide 1
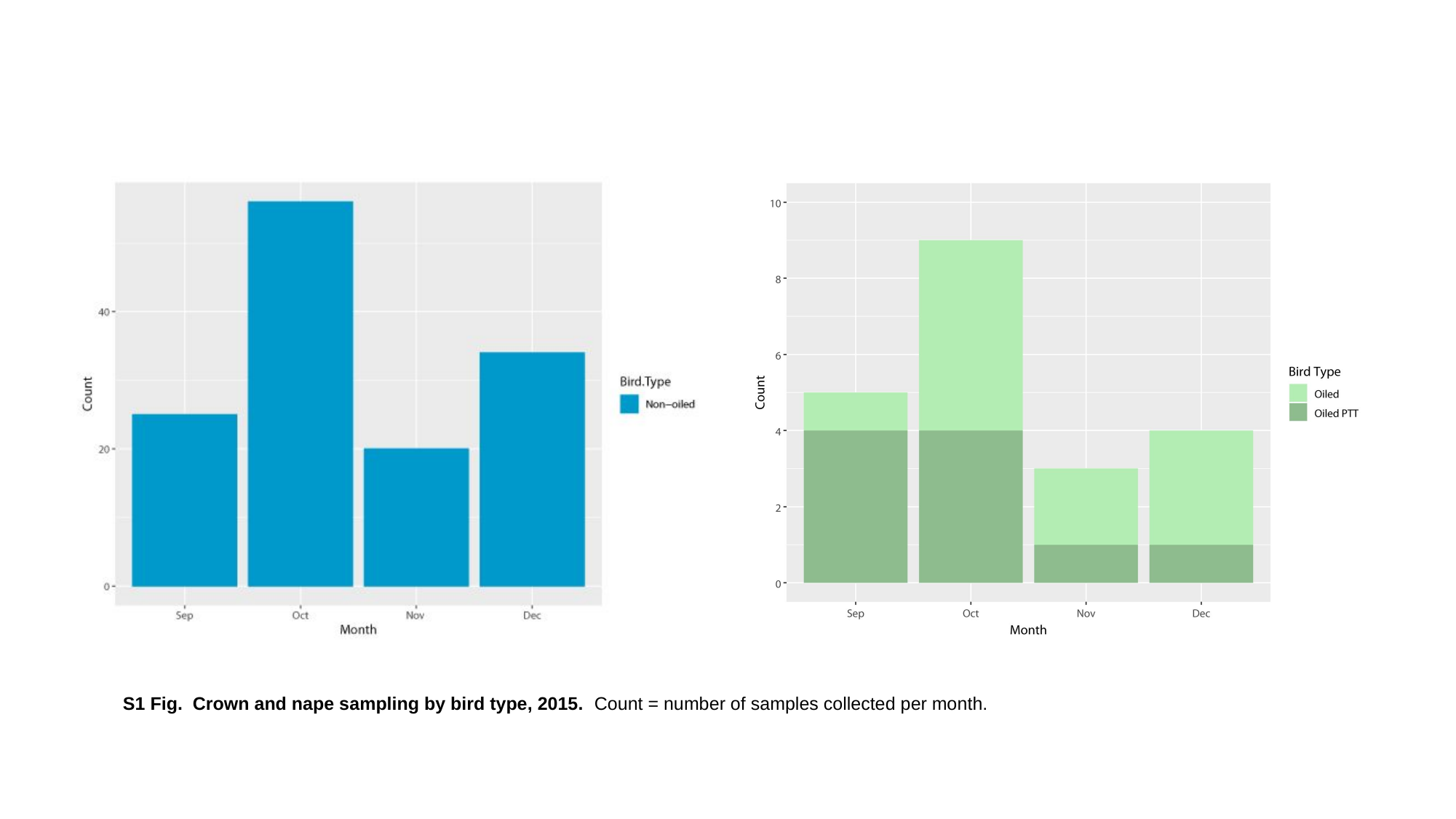

S1 Fig. Crown and nape sampling by bird type, 2015. Count = number of samples collected per month.

Supplement: S1 Fig — (PPTX) [file pone.0211932.s001.pptx]

## Slide 1
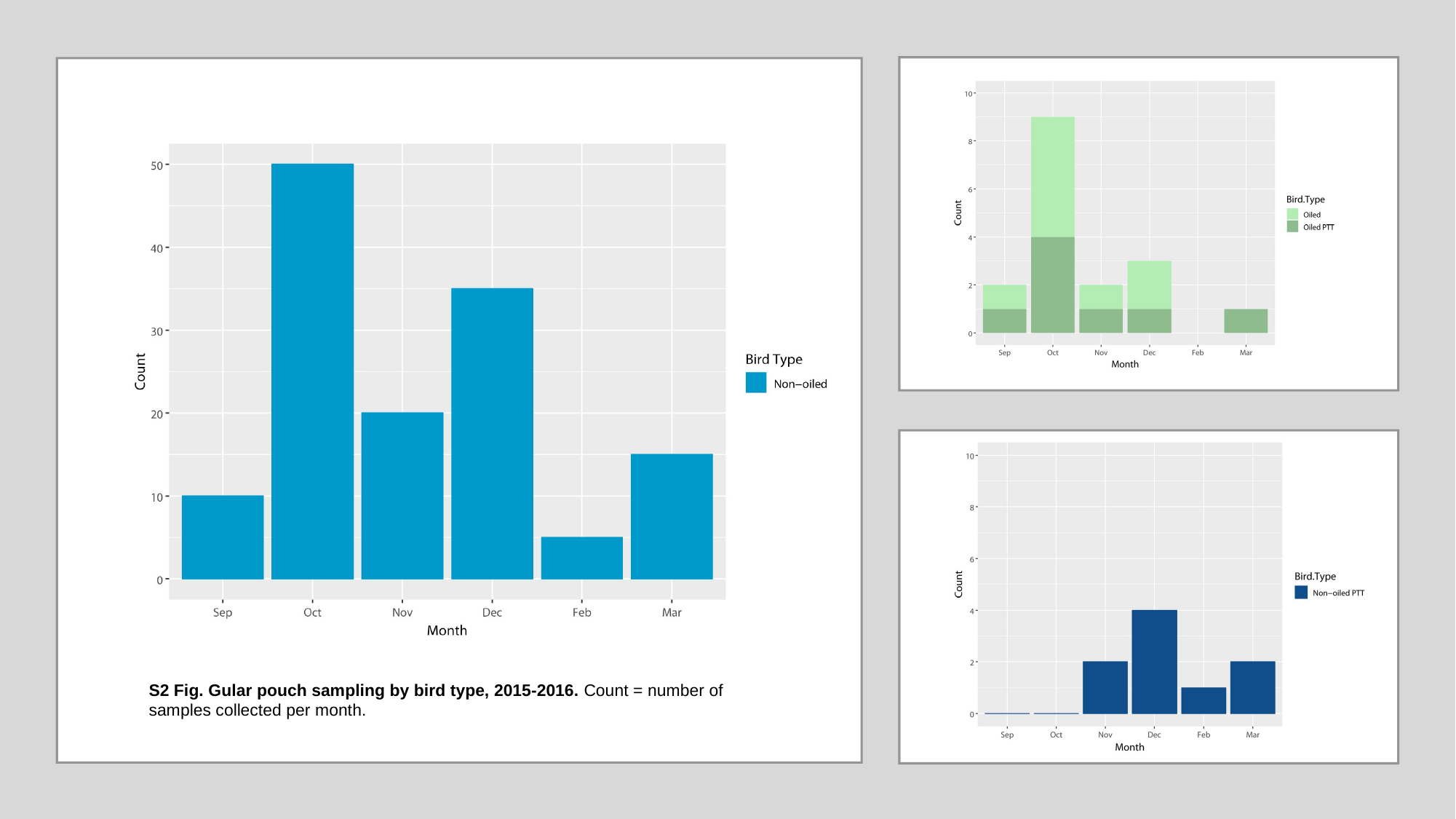

S2 Fig. Gular pouch sampling by bird type, 2015-2016. Count = number of samples collected per month.

Supplement: S2 Fig — (PPTX) [file pone.0211932.s002.pptx]
